# Blood pressure, pulse rate, and skin temperature during hot-water bathing in real-world settings among community-dwelling older adults: the HEIJO-KYO Study

**DOI:** 10.1265/ehpm.23-00320

**Published:** 2024-03-05

**Authors:** Yoshiaki Tai, Kenji Obayashi, Kazuki Okumura, Yuki Yamagami, Keigo Saeki

**Affiliations:** 1 Department of Epidemiology, Nara Medical University School of Medicine, Nara, Japan; 2 Department of Psychiatry, Nara Medical University School of Medicine, Nara, Japan

**Keywords:** Drowning, Cardiovascular diseases, Blood pressure, Heart rate, Water, Immersion, Temperature, Syncope, Consciousness

## Abstract

**Background:**

Home hot-tub bathing substantially increases drowning mortality rates among older adults in Japan. Previous laboratory studies on hemodynamic responses during hot-tub bathing have been inconsistent depending on the thermal conditions. Furthermore, real-world hemodynamic changes that occur during bathing remain poorly understood. This study investigated the association between individual thermal states and hemodynamic parameters during hot-tub bathing among community-dwelling older adults.

**Methods:**

In this cross-sectional study conducted between January 2016 and April 2019, which involved 1,479 older adults (median [range] age, 68 [40–90] years), skin temperature on the abdominal surface was measured every minute. Ambulatory blood pressure and pulse rate were recorded at 15-min intervals for 24 h. Participants underwent simultaneous living room temperature measurements in their homes, and the time and methods of bathing were recorded. Associations between skin temperature and hemodynamic parameters during bathing and between the pre-bath living room temperature and in-bath maximum proximal skin temperature were evaluated using mixed-effects and linear regression models, respectively.

**Results:**

A 1 °C increase in skin temperature was significantly associated with a 2.41 mmHg (95% confidence interval [CI]: 2.03–2.79) increase in systolic blood pressure and a 2.99 bpm (95% CI: 2.66–3.32) increase in pulse rate, after adjusting for potential confounders, including age, sex, body mass index, antihypertensive medication use, dyslipidemia, diabetes, and living room and outdoor temperatures. Significant interactions were not observed between sex and skin temperature in relation to systolic blood pressure and pulse rate (P = 0.088 and 0.490, respectively). One standard deviation lower living room temperature before bathing was significantly associated with a 0.41 °C (95% CI: 0.35–0.47) higher maximum skin temperature during bathing.

**Conclusions:**

Our findings suggest that pre-bath cold exposure may increase the skin temperature during hot-tub bathing, possibly resulting in excessive hemodynamic changes. This provides a framework for future interventions that utilize pre-bath thermal conditions and bathing environments to prevent bath-related deaths.

**Supplementary information:**

The online version contains supplementary material available at https://doi.org/10.1265/ehpm.23-00320.

## Background

In Japan, bathing in a bathtub for cleanliness and comfort within individual residences is a prevalent cultural practice [1]. The water temperature is maintained at approximately 41 °C, and the tub is typically deep enough to reach the neck [1, 2]. Depending on their age group, Japanese adults immerse themselves in hot water an average of 2.3–3.6 times per week during the summer and 4.6–4.9 times per week during the winter [3]. Bathing in hot water is associated with a decrease in nocturnal blood pressure [2], enhanced quality of sleep [4, 5], and a lower prevalence of depression [6]. Nevertheless, bath-related deaths hold significant public health importance in Japan, as other thermal conditions can also have impacts on health outcomes [7, 8]. The estimated number of bath-related deaths in Japan surpassed 13,000 during the winter (October 2012 to March 2013) and is expected to exceed 27,000 annually by 2035 due to the aging population [9]. Similarly, Japan has an extremely high drowning mortality rate among older adults, particularly linked to bathing in hot water, compared to other countries [10]. Therefore, effective prevention measures are needed.

Most bath-related deaths occur in winter (6.9 times more frequently than in summer), at home (94.3%), and among older adults (>90%) [11]. Although the mechanism underlying bath-related deaths is not fully understood, a previous study of autopsied cases reported that 79.1% of the cases showed signs of water inhalation [11]. Hyperthermia, cardiovascular diseases, stroke, and other conditions may cause bath-related deaths due to consciousness disturbances, weakness, and paralysis that lead to drowning. Furthermore, the transient loss of consciousness, including orthostatic hypotension and neurally mediated syncope induced by a reduction in hydrostatic pressure when standing in hot environments [12], can result in drowning in the absence of an underlying disease. Consequently, assessing hemodynamic changes induced by bathing is crucial.

Laboratory studies have examined hemodynamic changes during hot-water bathing. While the results consistently demonstrated an increase in pulse rate (PR) throughout the immersion [13, 14], the impact on blood pressure (BP) varied based on the age group and thermal bathing conditions [15–17]. Furthermore, these studies had relatively small sample sizes (up to 42 participants) and partially included young adults. Additionally, these settings do not account for individual preferences regarding bathing conditions or the surrounding environment.

This study evaluated hemodynamic parameters during hot-water bathing in real-world settings among community-dwelling older adults, reflecting individual bathing and surrounding environments. Additionally, the association between body surface temperature, which has been studied as an individual thermal state relating to health consequences [4, 18], and hemodynamic parameters during hot water immersion was quantified. This study also examined the relationship between the living room temperature before bathing and the peak body surface temperature during bathing to investigate whether the extent of passive body heating varies with the temperature of the participants’ environment before bathing.

We measured body surface temperature instead of water temperature in a bathtub because of the inverse relationship between water temperature and the duration of hot water immersion [2]. Therefore, measuring the maximum body surface temperature can be an improved method to assess the level of heat exposure compared to measuring water temperature in a bathtub. The representativeness of the study participants was evaluated by comparing the participant characteristics of the present study with those of a nationwide survey that used random sampling [19].

## Methods

### Participants and study protocol

This observational study obtained repeated measurements of BP, PR, and skin temperature for 24 hours (from noon on day 1 to noon on day 2) using data from the survey of a community-based cohort study. This survey was conducted between January 2016 and April 2019 and included 2,283 community-dwelling older adults. The participants were instructed to record the beginning and end times of bathing, duration of bathtub immersion, bedtime, and rising time in self-reported diaries. Ambulatory BP monitoring and actigraphy devices were attached to the dominant and non-dominant wrists, respectively. Additionally, temperature loggers were attached to the right lower quadrant of the abdominal surface and the non-dominant wrist to measure the proximal and distal skin temperatures, respectively. Actigraphy and distal skin temperature measurements were interrupted during bathing. Participants were eligible if they bathed during the survey period with proximal skin temperature measured during bathing, completed the self-reported diary, and had ≥10 daytime BP and PR recordings and ≥5 BP and PR recordings during bathing and the adjacent period (60 min before and after bathing) [15]. After excluding 469 participants who neither bathed nor showered, along with 20 lacking proximal skin temperature measurements during bathing, 98 with <10 daytime BP counts [20], and 211 with <5 BP counts during bathing and 1–60 min before and after bathing, 1,479 participants (median [range] age: 68 [40–90] years, 548 men and 931 women) were included in the analysis.

### Bathing activity records

The beginning and end times of bathing and the duration of bathtub immersion were determined using a self-reported diary. Bathing was defined as the time between entering and exiting the bathroom. Based on a previous study [15], the start time of bathtub immersion was defined as the time when the proximal skin temperature first exceeded 37 °C during bathing. Skin temperatures in the previous study rapidly increased just after bathtub immersion under different conditions, including in the bathing and dressing rooms at 10, 17.5 and 25.0 °C [15]. Participants were classified as follows: shower-only, if the diary indicated 0 min of bathtub immersion; warm bath immersion, ≥1 min of bathtub immersion without abdominal skin temperature exceeding 37 °C during bathing; and hot bath immersion, ≥1 min of bathtub immersion with abdominal skin temperature exceeding 37 °C during bathing [15, 21].

### BP and PR measurements

BP and PR were measured using a cuffless wrist-type ambulatory BP monitoring device (BPro, Healthstats International, Singapore) at 15-min intervals for 24 h, including the bathing period, using a waterproof cover for the device [22]. The BPro device captures the radial pulse wave reflection using modified applanation tonometry to calculate brachial BP [23]. The devices were calibrated by trained physicians using the average of the last five BP readings taken on the dominant arm while seated with an automated BP device (HEM-7200, OMRON Corp., Japan) and then placed on the radial artery on the dominant wrist to avoid attaching an actigraph on the dominant wrist. Daytime BP and PR were calculated as the means of BP and PR values during the out-of-bed period and evaluated using a self-reported diary. The double product (PR multiplied by systolic BP [SBP]) was also calculated as an indicator of myocardial oxygen demand. Changes in BP, PR, and the double product during bathing were evaluated using the difference between the daytime means of these metrics.

### Skin temperature measurements

In this study, proximal and distal skin temperatures were defined as the thermal measurements obtained from the abdominal surface area and distal region of the ventral forearm, respectively. Proximal and distal skin temperatures were measured at 1-min and 3-min intervals, respectively, using wireless temperature loggers (Thermochron iButton DS1992L; Maxim Integrated, CA, US). The accuracy, range, and resolution of the temperature logger were ±0.5 °C, −10 to +65 °C, and 0.0624 °C, respectively. The temperature logger was attached to the right upper quadrant of the abdominal surface using a Tegaderm transparent dressing (3M, St. Paul, MN, US). The logger for distal skin temperature was attached to the actigraph wristband using a plastic attachment, ensuring that the temperature logger sensor touched the ventral skin surface. The means of the proximal and distal skin temperatures during the daytime and 1–60 min before and after bathing were calculated based on the self-reported diary.

### Environmental temperature measurements

The indoor temperature was recorded at 10-min intervals using an identical temperature logger. The temperature logger was positioned 60 cm above the floor of each participant’s living room. Outdoor temperatures were obtained by consulting the meteorological station in the vicinity, using the address and survey date of each participant, at 10-min intervals. The means of the indoor and outdoor temperatures were calculated during the daytime and 1–60 min before and after bathing.

### Other measurements

Medical interviews confirmed smoking and drinking habits, household income, medical history, and medication use. The estimated glomerular filtration rate (eGFR) was determined using the equation recommended by the Japanese Society of Nephrology [24]. Diabetes was defined as a glycated hemoglobin level ≥6.5%, which is part of the clinical diagnosis criteria [25], or the use of pharmacological interventions. Dyslipidemia was defined as low-density lipoprotein cholesterol concentration ≥140 mg/dL, high-density lipoprotein cholesterol concentration <40 mg/dL, triglyceride concentration ≥150 mg/dL [26], or the use of pharmacological interventions. Physical activity was measured using actigraphy (GT3X-BT, ActiGraph LLC, Florida) with 1-min epochs, with the device worn on the non-dominant wrist. Mean physical activity counts were calculated during the daytime and 1–60 min before and after bathing.

### Statistical analysis

Descriptive characteristics are summarized as means (standard deviation [SD]), medians (interquartile range), and counts (percentage) for normally distributed continuous, non-normally distributed continuous, and categorical variables, respectively. Mean values, median values, and proportions were compared utilizing Welch’s t-test, Mann–Whitney U test, and χ^2^ test, respectively. Using the Z-test, the mean body mass index (BMI) and eGFR of the participants were compared with those of a nationwide survey conducted in 2016 (the first year of this study) and the following year, respectively, since eGFR was not reported in 2016.

We used a generalized additive model to analyze time-dependent variations in hemodynamic parameters (SBP, diastolic BP [DBP], PR, and the double product) before and after the beginning of bathing, as well as the associations between proximal skin temperature and hemodynamic parameters. In these models, the hemodynamic parameters were centered as deviations from each participant’s daytime mean. To evaluate differences in the association between proximal skin temperature and hemodynamic parameters based on the bathing method and environment, these models were applied separately to individuals who took a bath with low-to-moderate heat intensity (shower and warm bath immersion) and high heat intensity (hot bath immersion) and those who bathed under colder or warmer outdoor temperatures. These models were also applied for the age-quartile groups, sexes, and the four seasons. The survey-year solstices and equinoxes were used to define the four seasons.

The associations between proximal skin temperature and hemodynamic parameters during bathing and the adjacent period (1–60 min before and after bathing) were examined using a linear mixed-effects model (random intercepts and fixed coefficients) among participants who underwent hot bath immersion. Based on the plots in Fig. 2, the dataset was divided into two groups based on whether the proximal skin temperature was ≥36 °C or <36 °C. The variance-covariance matrices were specified to be unstructured. The model parameters were estimated using restricted maximum likelihood. The adjusted model included age (years), sex, BMI (kg/m^2^), alcohol intake (≥30 g/day), current smoking, household income (≥4 million Japanese Yen/year), antihypertensive medication use, dyslipidemia, diabetes, eGFR (mL/minute/1.73 m^2^), and the means of indoor temperature (°C), outdoor temperature (°C), and physical activity 1–60 min before and after bathing (vector magnitude [VM] count/min). Interaction terms were introduced into the same model to test for the effect modification of sex and skin temperatures.

We used multivariable linear regression models to examine the association between the mean environmental and skin temperatures 1–60 min before bathing and the maximum proximal skin temperature during bathing and between these temperatures and the duration of bathtub immersion. Interaction terms were introduced into the same model to test for the effect of modifying environmental and skin temperatures. The adjusted models included age (years), sex, BMI (kg/m^2^), alcohol intake (≥30 g/day), current smoking status, household income (≥4 million Japanese Yen/year), antihypertensive medication use, dyslipidemia, diabetes, eGFR (mL/minute/1.73 m^2^), and mean physical activity 1–60 min before bathing (VM count/min). The goodness of fit was evaluated using R^2^. Adjusted R^2^ was calculated for a multivariable linear regression model. Marginal and conditional R^2^, which represent variance explained by the fixed effects and the entire model, respectively, were calculated for a mixed-effect model. We did not apply imputations for missing data in our analysis, as the proportion of missing data was negligible (1.5%) [27]. The numbers of participants whose data were missing for particular variables are described in the footnotes of Tables 2 and 3.

All analyses were performed using the R software, version 4.1.2. Intraclass correlation, mixed-effect model, generalized additive model analyses, and R^2^ for the mixed-effect model were performed using R packages ICC, lmerTest, mgcv, and MuMIn [28–32], respectively. All *P*-values were two-sided, and *P*-values <0.05 were considered statistically significant.

## Results

Table 1 presents participant characteristics categorized by the bathing method. The proportion of current smokers in the hot bath immersion group was significantly lower than those in the shower and warm bath groups. The hot bath immersion group had significantly lower proximal and distal skin temperatures and lower environmental temperatures 1–60 min before bathing than the shower and warm bath groups. While the SBP before bathing did not differ significantly between the two groups, the SBP during bathing was significantly higher in the hot bath immersion group than in the shower and warm bath groups (difference 3.39 mmHg, 95% confidence interval [CI] 0.42–6.36). The mean duration of bathtub immersion was 11.9 (SD, 6.2) min in the hot bath immersion group. The mean BMI and eGFR of the participants did not differ significantly from those of the participants aged ≥40 years in the nationwide survey that used stratified random sampling (P = 0.826 and P = 0.169, respectively, in men; P = 0.153 and P = 0.493, respectively, in women).

**Table 1:** Participant characteristics stratified by bathing method

	**Hot bath immersion**	**Shower or warm bath immersion**	***P* value**
	**(n = 1,169)**	**(n = 310)**	
Basic characteristics			
Age, mean (SD), years	67.8 (7.9)	67.6 (7.8)	0.575
Male, n	420 (35.9%)	128 (41.3%)	0.082
Current smoker, n	58 (5.0%)	26 (8.4%)	0.021
Alcohol consumption (≥30 g/day), n	140 (12.0%)	43 (13.9%)	0.368
Household income (≥4 million JPY per year), n	488 (41.9%)	127 (41.6%)	0.946
Body mass index, mean (SD), kg/m^2^	23.0 (3.0)	23.1 (3.0)	0.826
Antihypertensives use, n	426 (36.4%)	104 (33.7%)	0.364
Dyslipidemia, n	377 (32.2%)	121 (39.0%)	0.025
Diabetes, n	68 (5.8%)	20 (6.5%)	0.675
eGFR, mean (SD), mL/min/1.73 m^2^	71.3 (13.4)	72.2 (13.5)	0.262
Bathing related factors			
Start time of bathing, mean (SD), clock time	21:06 (1:45)	20:51 (2:19)	0.042
Time spent in bathroom, mean (SD), min	26.1 (9.6)	20.1 (9.8)	<0.001
Proximal skin temperature, mean (SD), °C			
1–60 min before bathing	34.8 (1.2)	34.9 (1.2)	0.036
Maximum during bathing	39.4 (1.1)	35.7 (1.4)	<0.001
Distal skin temperature, mean (SD), °C			
1–60 min before bathing	32.8 (1.9)	33.6 (1.7)	<0.001
Environmental temperature, mean (SD), °C			
Indoor, 1–60 min before bathing	21.1 (4.7)	24.7 (5.1)	<0.001
Outdoor, 1–60 min before bathing	13.7 (8.4)	20.0 (9.4)	<0.001
Systolic blood pressure, mean (SD), mmHg			
1–60 min before bathing	121.8 (16.5)	122.6 (18.3)	0.464
During bathing activity	127.0 (17.3)	123.6 (19.3)	0.025
Diastolic blood pressure, mean (SD), mmHg			
1–60 min before bathing	71.8 (11.1)	72.3 (11.8)	0.547
During bathing activity	74.2 (11.5)	73.1 (12.1)	0.258
Pulse rate, mean (SD), beats per min			
1–60 min before bathing	69.5 (9.8)	70.0 (10.3)	0.419
During bathing activity	74.7 (10.7)	73.1 (10.9)	0.075
Physical activity, mean (SD), VM counts/min			
1–60 min before bathing	2232 (1176)	2249 (1258)	0.817

P-values were calculated using Welch’s t-test and χ^2^ test.

eGFR, estimated glomerular filtration rate; JPY, Japanese Yen; SD, standard deviation; VM, vector magnitude

Hot- and warm bath immersions were defined as bathtub immersions in which the proximal skin temperature reached or did not reach 37 °C.

**Fig. 1:**
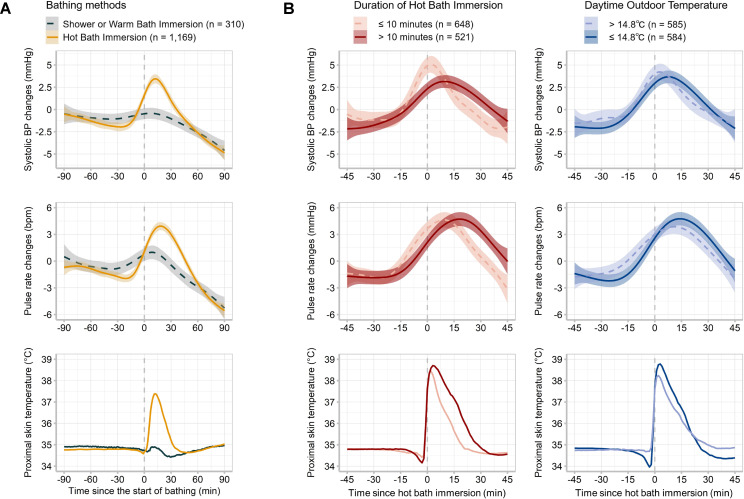
Time-dependent changes in systolic blood pressure, pulse rate, and proximal skin temperature during bathing activity A generalized additive model was used for time-dependent changes in systolic blood pressure and pulse rate measured at 15-min intervals. The shaded areas indicate the upper and lower 95% confidence intervals. A line graph was used for time-dependent changes in the proximal skin temperature measured every minute. The x-axis displays (A) the time since entering the bathroom and (B) the time since the beginning of the hot bath immersion. Blood pressure and pulse rate were centered within the individual daytime means. bpm, beats per minute

Figure 1A depicts the time-dependent changes in SBP, PR, and proximal skin temperature 90 min before and after the start of bathing. The hot bath immersion group showed a greater increase in SBP and PR after bathing than the shower or warm bath immersion groups. Figure 1B illustrates the changes in SBP, PR, and proximal skin temperature observed 45 min before and after immersion commencement. Individuals who had hot bath immersions for ≤10 min tended to have a high and early SBP peak (5.07 [95% CI: 4.13–6.01] mmHg, 1.9 min after immersion commencement) compared with those who had hot baths for >10 min (3.15 [95% CI: 2.43–3.87] mmHg, 9.6 min after immersion commencement). Although the peaks in PR change were similar between the same two groups (4.63 [95% CI: 3.76–5.50] vs. 4.72 [95% CI: 3.93–5.50] bpm), the peak time for individuals who had hot bath immersions for ≤10 min (9.4 min after immersion commencement) tended to be earlier than that for those who had hot bath immersions for >10 min (18.2 min after immersion commencement). Peak SBP and PR changes were not notably distinct between the participants who completed the survey under cold and warm outdoor temperatures (Fig. 1B). Time-dependent DBP and double product changes at the beginning of bathing and hot water immersion are presented in Additional file 1.

**Fig. 2:**
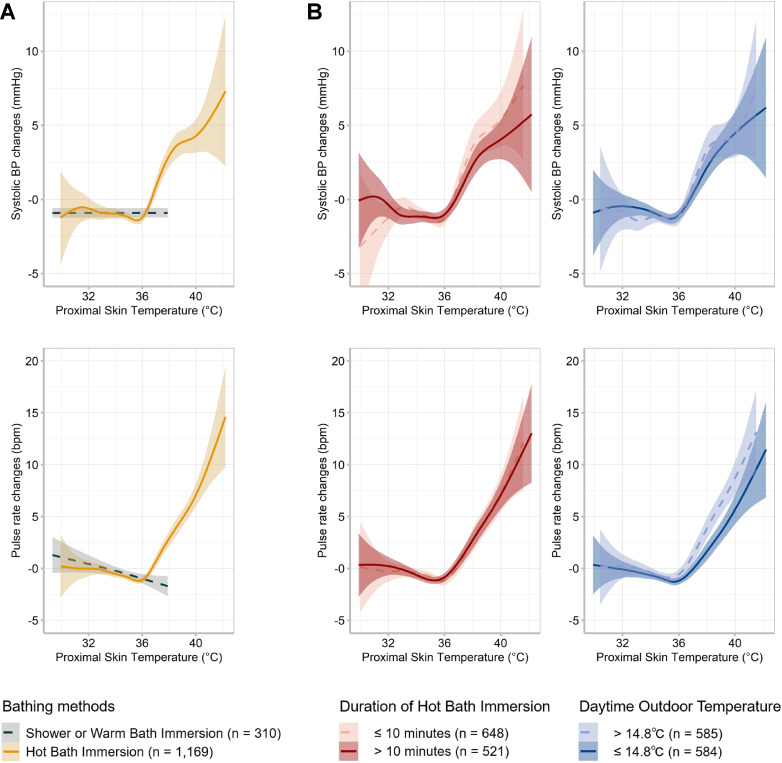
Association between proximal skin temperature and hemodynamic parameters during bathing and the adjacent period Participants were divided into two groups based on bathing method (A), duration of hot bath immersion (red line in B), and median daytime outdoor temperature (blue line in B). A generalized additive model was used to determine the association between proximal skin temperature and systolic BP/pulse rate. The shaded areas indicate the upper and lower 95% confidence intervals. Systolic BP, pulse rate, and proximal skin temperature were measured during bathing and 1–60 min before and after bathing. Blood pressure and pulse rate were centered within the individual daytime means. BP, blood pressure; bpm, beats per minute

The associations between proximal skin temperature and hemodynamic parameters observed during bathing and 1–60 min before and after bathing are shown in Fig. 2 and Additional files 2 and 3. In the hot bath immersion group, the associations between proximal skin temperature and hemodynamic parameters were positive for proximal skin temperatures ≥36 °C but negative for temperatures <36 °C (Fig. 2A and Table 2). In the shower and warm bath immersion groups, a positive association was not observed between proximal skin temperature and hemodynamic parameters. For proximal temperatures ≥36 °C, the positive associations remained unchanged when participants were divided into median groups by hot bath immersion duration or outdoor temperature (Fig. 2B). The variations in the positive correlations between the age-quartile groups, sexes, and the four seasons remained indistinct (Additional file 3). For proximal skin temperatures <36 °C, weak negative associations were observed between proximal skin temperature and hemodynamic parameters in the hot bath immersion group (Fig. 2A, Additional file 2).

**Table 2:** Association between proximal skin temperature and hemodynamic parameters among participants who underwent hot bath immersion

**Variables**	**Number of** **participants**	**Number of** **measurements**	**Crude**		Adjusted*	
	
			**Coefficient** **(95% CI)**	***P* value**	**Coefficient** **(95% CI)**	***P* value**
**per 1 °C increase from 36 °C**						
Systolic BP, mmHg	833	2,343	2.46 (2.09, 2.83)	<0.001	2.41 (2.03, 2.79)	<0.001
Diastolic BP, mmHg	833	2,343	1.22 (1.03, 1.40)	<0.001	1.18 (1.00, 1.37)	<0.001
Pulse rate, bpm	833	2,343	3.05 (2.73, 3.38)	<0.001	2.99 (2.66, 3.32)	<0.001
Double product, mmHg·bpm	833	2,343	556 (498, 613)	<0.001	544 (485, 602)	<0.001
**per 1 °C increase below 36 °C**						
Systolic BP, mmHg	1,150	8,301	−1.14 (−1.38, −0.90)	<0.001	−1.08 (−1.32, −0.84)	<0.001
Diastolic BP, mmHg	1,150	8,301	−0.57 (−0.68, −0.45)	<0.001	−0.55 (−0.67, −0.43)	<0.001
Pulse rate, bpm	1,150	8,301	−1.30 (−1.52, −1.09)	<0.001	−1.26 (−1.48, −1.04)	<0.001
Double product, mmHg·bpm	1,150	8,301	−243 (−281, −205)	<0.001	−229 (−267, −191)	<0.001

Coefficients were analyzed using a linear mixed model. There were fewer than 1,169 participants in the two skin temperature categories because a certain number of the participants did not have a BP/PR reading while their skin temperature was ≥36 or <36 °C. *Adjusted for age (years), sex, body mass index (kg/m^2^), alcohol intake (≥30 g/day), current smoking, household income (≥4 million Japanese Yen/year), antihypertensives use, dyslipidemia, diabetes, estimated glomerular filtration rate (mL/min/1.73 m^2^), and the means of indoor temperature (°C), outdoor temperature (°C), and physical activity 1–60 min before and after bathing (vector magnitude count/min). The number of participants whose values were missing for household income, antihypertensive medication use, and mean indoor temperature 1–60 min before and after bathing were 8, 1, and 13, respectively.

BP, blood pressure; bpm, beats per minute; CI, confidence interval; Ref, reference

Linear mixed-effects model analysis revealed that as proximal skin temperature exceeded 36 °C, it was positively associated with hemodynamic parameters (Table 2). A 1 °C increase in proximal skin temperature was significantly associated with a 2.41 mmHg (95% CI: 2.03–2.79, marginal R^2^ = 0.09, conditional R^2^ = 0.82) increase in SBP, 1.18 mmHg (95% CI: 1.00–1.37, marginal R^2^ = 0.09, conditional R^2^ = 0.91) increase in DBP, 2.99 bpm (95% CI: 2.66–3.32, marginal R^2^ = 0.13, conditional R^2^ = 0.63) increase in PR, and 544 (95% CI: 485–602, marginal R^2^ = 0.13, conditional R^2^ = 0.66) increase in the double product, after adjusting for age, sex, BMI, alcohol intake, current smoking, household income, antihypertensive medication use, dyslipidemia, diabetes, eGFR, indoor and outdoor temperature, and physical activity. In the same model, significant interactions between sex and proximal skin temperature in relation to systolic BP and PR were not observed (P = 0.088 and 0.490, respectively). A 1 °C decrease in proximal skin temperature within the range <36 °C was significantly associated with a 1.08 mmHg (95% CI: 0.84–1.32, marginal R^2^ = 0.07, conditional R^2^ = 0.80) increase in SBP, 0.55 mmHg (95% CI: 0.43–0.67, marginal R^2^ = 0.08, conditional R^2^ = 0.89) increase in DBP, 1.26 bpm (95% CI: 1.04–1.48, marginal R^2^ = 0.07, conditional R^2^ = 0.58) increase in PR, and 229 (95% CI: 191–267, marginal R^2^ = 0.06, conditional R^2^ = 0.64) increase in the double product, after adjusting for the same variables.

The means of the outdoor, indoor, and distal skin temperatures 1–60 min before bathing were negatively associated with the maximum values of the proximal skin temperatures during bathing and the duration of bathtub immersion (Table 3). In the multivariable linear regression model, 1 SD lower outdoor, indoor, and distal skin temperatures 1–60 min before bathing were significantly associated with 0.43 °C (95% CI: 0.37–0.49, adjusted R^2^ = 0.18), 0.41 °C (95% CI: 0.35–0.47, adjusted R^2^ = 0.16), and 0.24 °C (95% CI: 0.18–0.30, adjusted R^2^ = 0.07) higher maximum proximal skin temperatures during bathing, respectively. In the same linear regression model, the outdoor and indoor temperatures and indoor and distal skin temperatures significantly interacted with one another (P = 0.007 and P = 0.003, respectively). In contrast, the mean proximal skin temperature 1–60 min before bathing was not significantly associated with the maximum proximal skin temperature during bathing. In the adjusted model, 1 SD lower outdoor, indoor, and distal skin temperatures 1–60 min before bathing were significantly associated with 1.59 (95% CI: 1.24–1.94, adjusted R^2^ = 0.07), 1.64 (95% CI: 1.29–1.64, adjusted R^2^ = 0.08), and 1.44 (95% CI: 1.08–1.80, adjusted R^2^ = 0.06) min of duration of bathtub immersion, respectively. However, a significant interaction was not observed regarding the duration of bathtub immersion.

**Table 3:** Potential influence of thermal conditions before bathing on proximal skin temperature during hot bath immersion

**Variables**	**Maximum proximal skin temp** **during immersion (°C)**			**Duration of hot water immersion** **(minutes)**		

	**Coefficient**	**(95% CI)**	***P* value**	**Coefficient**	**(95% CI)**	***P* value**
Crude (per SD increase in temp [°C]*)						
Outdoor temp	−0.43	(−0.48, −0.37)	<0.001	−1.67	(−2.01, −1.33)	<0.001
Indoor temp	−0.41	(−0.47, −0.36)	<0.001	−1.71	(−2.05, −1.37)	<0.001
Distal skin temp	−0.25	(−0.31, −0.19)	<0.001	−1.39	(−1.74, −1.03)	<0.001
Proximal skin temp	0.02	(−0.04, 0.08)	0.568	−0.02	(−0.38, 0.33)	0.904
Interaction terms						
Indoor × Outdoor temps	−0.07	(−0.13, −0.01)	0.035	−0.35	(−0.73, 0.04)	0.078
Distal skin × Indoor temp	−0.09	(−0.14, −0.03)	0.004	0	(−0.35, 0.35)	0.995
Proximal skin temp × Indoor temp	0	(−0.06, 0.06)	0.963	0.08	(−0.27, 0.43)	0.652

Adjusted† (per SD increase in temp [°C]*)						
Outdoor temp	−0.43	(−0.49, −0.37)	<0.001	−1.59	(−1.94, −1.24)	<0.001
Indoor temp	−0.41	(−0.47, −0.35)	<0.001	−1.64	(−1.98, −1.29)	<0.001
Distal skin temp	−0.24	(−0.30, −0.18)	<0.001	−1.44	(−1.80, −1.08)	<0.001
Proximal skin temp	0.02	(−0.05, 0.09)	0.582	−0.17	(−0.55, 0.22)	0.392
Interaction terms						
Indoor temp × Outdoor temp	−0.09	(−0.15, −0.02)	0.008	−0.30	(−0.69, 0.09)	0.138
Distal skin temp × Indoor temp	−0.10	(−0.15, −0.04)	<0.001	0.02	(−0.32, 0.37)	0.896
Proximal skin temp × Indoor temp	0.01	(−0.05, 0.07)	0.754	0.33	(−0.02, 0.69)	0.063

Linear regression model was used to calculate the regression coefficients.

*Each temperature represents the mean temperature 1–60 min before bathing.

†Adjusted for age (years), sex, body mass index (kg/m^2^), alcohol intake (≥30 g/day), current smoking, household income (≥4 million Japanese Yen/year), antihypertensives use, dyslipidemia, diabetes, estimated glomerular filtration rate (mL/min/1.73 m^2^), and mean physical activity 1–60 min before bathing (vector magnitude count/min). The number of missing values in household income, antihypertensive medication use, and mean of indoor temperature 1–60 min before and after bathing were 8, 1, and 13, respectively.

CI, confidence interval; SD, standard deviation, temp; temperature

## Discussion

BP, PR, and the double product increased during hot bath immersion in real-world settings among community-dwelling older adults. Additionally, we quantified changes in these hemodynamic parameters per 1 °C increase or decrease from 36 °C in proximal skin temperature during bathing and the adjacent period, adjusting for potential confounders, including age, sex, smoking and drinking habits, coexisting conditions, and environmental temperatures. Moreover, we showed that indoor, outdoor, and distal skin temperatures before bathing were negatively associated with the maximum proximal skin temperature during bathing. In this analysis, a significant interaction was observed between the outdoor and indoor temperatures and between the indoor and distal skin temperatures. To our knowledge, this is the first study to demonstrate an increase in BP, PR, and their double product during bathing in relation to environmental and skin temperatures under free-living conditions.

These results are consistent with those of previous studies that investigated hemodynamic responses during hot-water bathing in laboratory settings. Previous research revealed that, in older adults, SBP and PR increased by approximately 10 mmHg and 14–20 bpm, respectively, immediately after hot bath immersion when the water temperature was at ≥41 °C [13, 14]. In contrast, bathing in a tub with water temperatures ≤39 °C did not increase BP or PR [14]. Although we did not measure the bathtub water temperature of each participant’s home, a higher proximal skin temperature during bathing, which should reflect higher water temperature in a bathtub, was associated with higher BP and PR.

Our observations provide novel insights into the relationship between hot bath immersion and hemodynamic responses concerning pre-bath thermal conditions. Our results showed that lower indoor and distal skin temperatures before bathing were associated with a higher maximum proximal skin temperature during bathing and a longer duration of immersion. Higher maximum proximal skin temperature during bathing could lead to a greater increase in BP, PR, and their double product. Additionally, significant interactions were observed between outdoor and indoor temperatures before bathing and between indoor and distal skin temperatures before bathing, affecting the maximum proximal skin temperature during bathing. Environmental or behavioral factors can account for these findings, which implies that even in cold seasons and in cold indoor environments, individuals do not need to bathe with higher levels of heat exposure when they are in warmer indoor environments or have warmer body surface temperatures, respectively. These results suggest that during the cold season, when more bath-related deaths occur, a warm indoor environment or warm individual microclimate before bathing may prevent excessive heat exposure during bathing, which leads to a higher proximal skin temperature and higher BP, PR, and their double product.

Possible explanations for the BP and PR elevation during bathing include increased sympathetic activity and heat-dissipating responses. A physiological study demonstrated increased sympathetic nerve activity and plasma catecholamine concentration during whole-body passive heating in young and aged individuals [33]. Additionally, a rapid increase in body surface temperature or hot baths with water temperatures ≥42 °C can trigger nociceptive inputs via the transient receptor potential cation channel vanilloid subfamily member 1 [34, 35], leading to increase in sympathetic activity [36]. Regarding PR, passive heat stress causes increases in cardiac output, primarily through an elevated heart rate, to compensate for the redistribution of blood from central to peripheral circulation [37]. Moreover, our results indicated that BP and PR increased before bathing (Fig. 1), suggesting that the physical exertion required to undress and the cold exposure caused by undressing may affect BP and PR before bathtub immersion [18, 38].

The clinical implication of these findings is that interventions targeting indoor temperature and individual thermal conditions before bathing can contribute to creating a safer bathing environment, thereby reducing the risk of bath-related deaths. Physiological changes that occur during bathing, including short-term variations in BP and PR, an increase in myocardial oxygen demand, and hyperthermia, can precipitate the onset of cardiovascular disease, stroke, and transient loss of consciousness [12, 39, 40], possibly resulting in bathtub drowning. To avoid these consequences, a Japanese expert committee has recommended that older adults bathe for ≤10 min at a water temperature ≤40 °C [41]. Nevertheless, attaining these objectives may be challenging without properly regulating indoor or individual thermal conditions before bathing.

The curves generated using the GAM exhibit no discernible differences when evaluating the associations between proximal skin temperature and systolic blood pressure/PR across the age groups, sexes, and seasons. Inadequate sample sizes in the higher range of proximal skin temperatures could explain these results. Physiological experiments could detect these differences across age groups, sexes, and seasons in controlled and forced settings. The maximum proximal skin temperature of each participant during bathing may be determined by the environmental temperature or participant’s preference, which is inherent to observational studies.

A strength of this study is the measurement of BP, PR, and skin temperature during bathing in real-world settings, which enabled the analysis of the relationship between these physiological factors within the bathing conditions selected by individual participants.

This study had several limitations. First, the cross-sectional design limits further investigations into the temporal relationship between proximal skin temperature and hemodynamic parameters. Nonetheless, a short-term longitudinal relationship was observed between pre-bath thermal conditions and the maximum proximal skin temperature during bathing. Second, the participants were not randomly selected, potentially introducing selection bias. However, the participants’ mean BMI and eGFR were approximately comparable to those of a national survey based on stratified random sampling [19]. Third, the hemodynamic parameters were assessed at 15-min intervals. Consequently, a few participants with no BP or PR readings while bathing were excluded. Moreover, the highest BP and PR values for each participant during bathing based on the measurements collected every minute could not be determined. Nevertheless, the relationship between pre-bath thermal conditions and maximum proximal skin temperature based on measurements at 1-min intervals was investigated, assuming that the highest proximal skin temperature would be a surrogate for the highest BP and PR. Fourth, water and core body temperature measurements were not conducted to assess heat exposure and its impact on individuals. Nevertheless, the feasibility of using core body temperature measurements in real-world scenarios as a preventive measure for bath-related deaths may be hindered by the invasive nature of this approach. Fifth, the occurrence of transient arrhythmia during bathing was unconfirmed, possibly leading to erroneous BP and PR measurements. Passive body heating can trigger an immune response through an increase in immune cells and cortisol levels [42], which could result in an inflammatory-mediated transient arrhythmia [43]. Finally, transient loss of consciousness or onset of cardiovascular disease, stroke, or other conditions was not observed during this survey. Consequently, the findings were based on the physiological range of the hemodynamic parameters.

## Conclusions

This study revealed increases in BP, PR, and double product during hot bath immersion in real-life settings among older adults, the population at greatest risk of bath-related deaths. The study revealed a positive association between proximal skin temperature and hemodynamic parameters, including BP, PR, and their double product, during hot bath immersion. Pre-bath thermal conditions, including outdoor, indoor, and distal proximal skin temperatures, were indicated to influence the maximum proximal skin temperature during hot water immersion and the duration of bathtub immersion. These findings provide a framework for future interventions that utilize pre-bath thermal conditions and bathing environments to prevent bath-related deaths.

## Abbreviations

BMI: body mass index
BP: blood pressure
BPM: beats per minute
CI: confidence interval
DBP: diastolic blood pressure
eGFR: estimated glomerular filtration rate
PR: pulse rate
SBP: systolic blood pressure
SD: standard deviation
VM: vector magnitude

## Supplementary information

**Additional file 1.** Time-dependent changes in diastolic blood pressure, double product, and proximal skin temperature during bathing. **Additional file 2.** Association of proximal skin temperature with diastolic blood pressure and double product during bathing activity. **Additional file 3.** Association between proximal skin temperature and hemodynamic parameters during bathing and the adjacent period divided by four seasons, sex, and age groups.

## Declarations

### Availability of data and materials

Data are available from the corresponding author upon reasonable request.
