# Supplementary material for: Blood pressure, pulse rate, and skin temperature during hot-water bathing in real-world settings among community-dwelling older adults: the HEIJO-KYO Study
Source: Environ Health Prev Med. 2024 Mar 5;29:12. doi: 10.1265/ehpm.23-00320 (PMC10937243; doi:10.1265/ehpm.23-00320)
Supplement: Supplementary file 1 — Additional file 1. Time-dependent changes in diastolic blood pressure, double product, and proximal skin temperature during bathing. Additional file 2. Association of proximal skin temperature with diastolic blood pressure and double product during bathing activity. Additional file 3. Association between proximal skin temperature and hemodynamic parameters during bathing and the adjacent period divided by four seasons, sex, and age groups. [file ehpm-29-012-s001.docx]

**SUPPLEMENTAL MATERIALS**

Supplement to “*Blood Pressure, Pulse Rate, and Skin Temperature during Hot-Water Bathing in Real-World Settings among Community-Dwelling Older Adults: the HEIJO-KYO study”*

Yoshiaki Tai, Kenji Obayashi, Kazuki Okumura, Yuki Yamagami, Keigo Saeki

**Correspondence**

Yoshiaki Tai

Department of Epidemiology, Nara Medical University School of Medicine

Phone number: +81-744-29-8841

Fax number: +81-744-29-0673

Email: yoshiaki.t@naramed-u.ac.jp

**LIST OF SUPPLEMENTAL CONTENTS**

**Additional file 1**. Time-dependent changes in diastolic blood pressure, double product, and proximal skin temperature during bathing

**Additional file 2**. Association of proximal skin temperature with diastolic blood pressure and double product during bathing activity

**Additional file 3**. Association between proximal skin temperature and hemodynamic parameters during bathing and the adjacent period divided by four seasons, sex, and age groups

**Additional file 1**. Time-dependent changes in diastolic blood pressure, double product, and proximal skin temperature during bathing and the adjacent period.


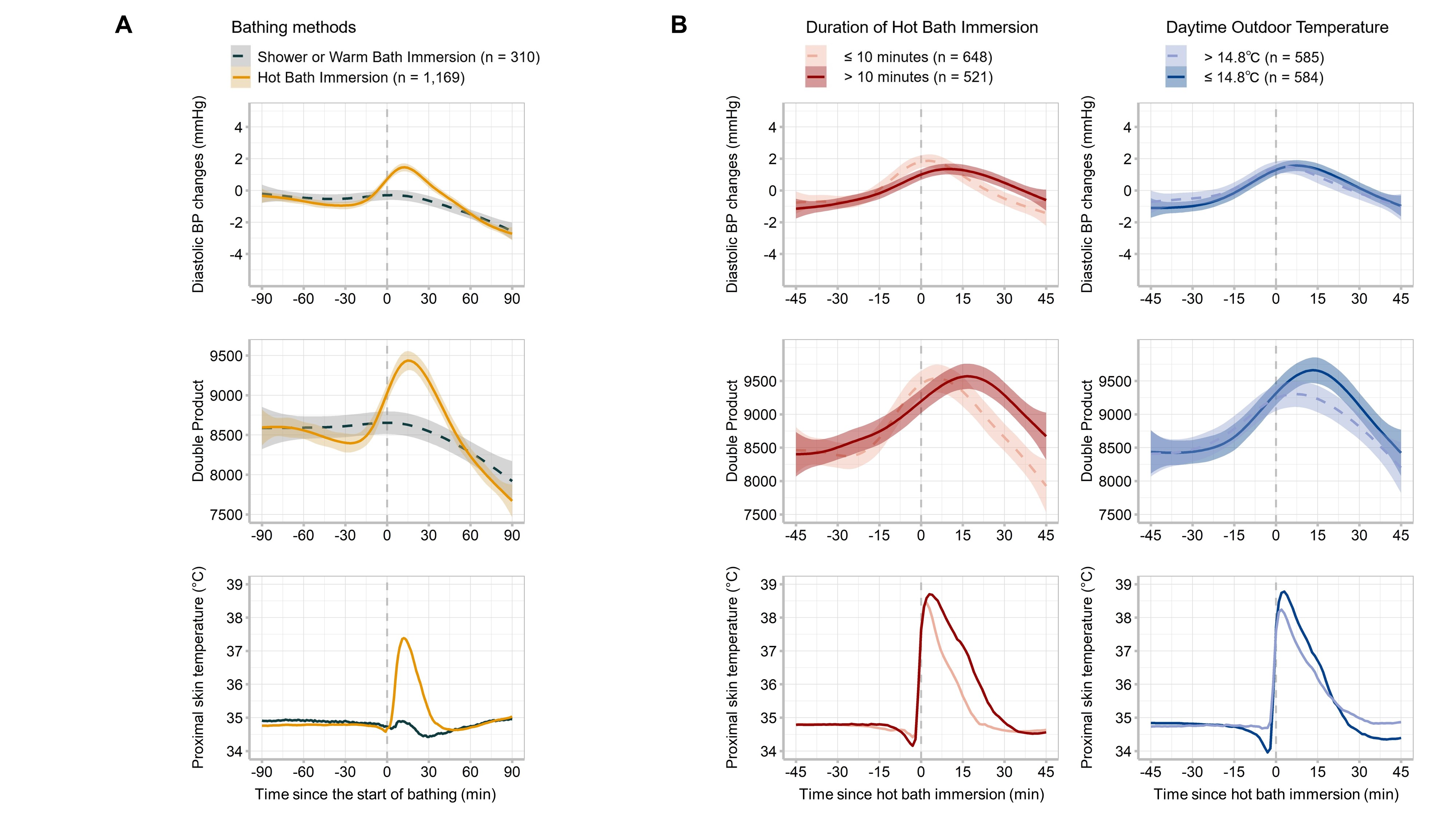


A generalized additive model was used for time-dependent changes in diastolic blood pressure and double product measured at 15-min intervals. The shaded areas indicate the upper and lower 95% confidence intervals. A line graph was used for time-dependent changes in the proximal skin temperature measured every minute.

The x-axis displays the time since entering the bathroom (A) and the time since the beginning of the hot bath immersion (B). Blood pressure was centered within the individual daytime mean.

bpm, beats per minute.


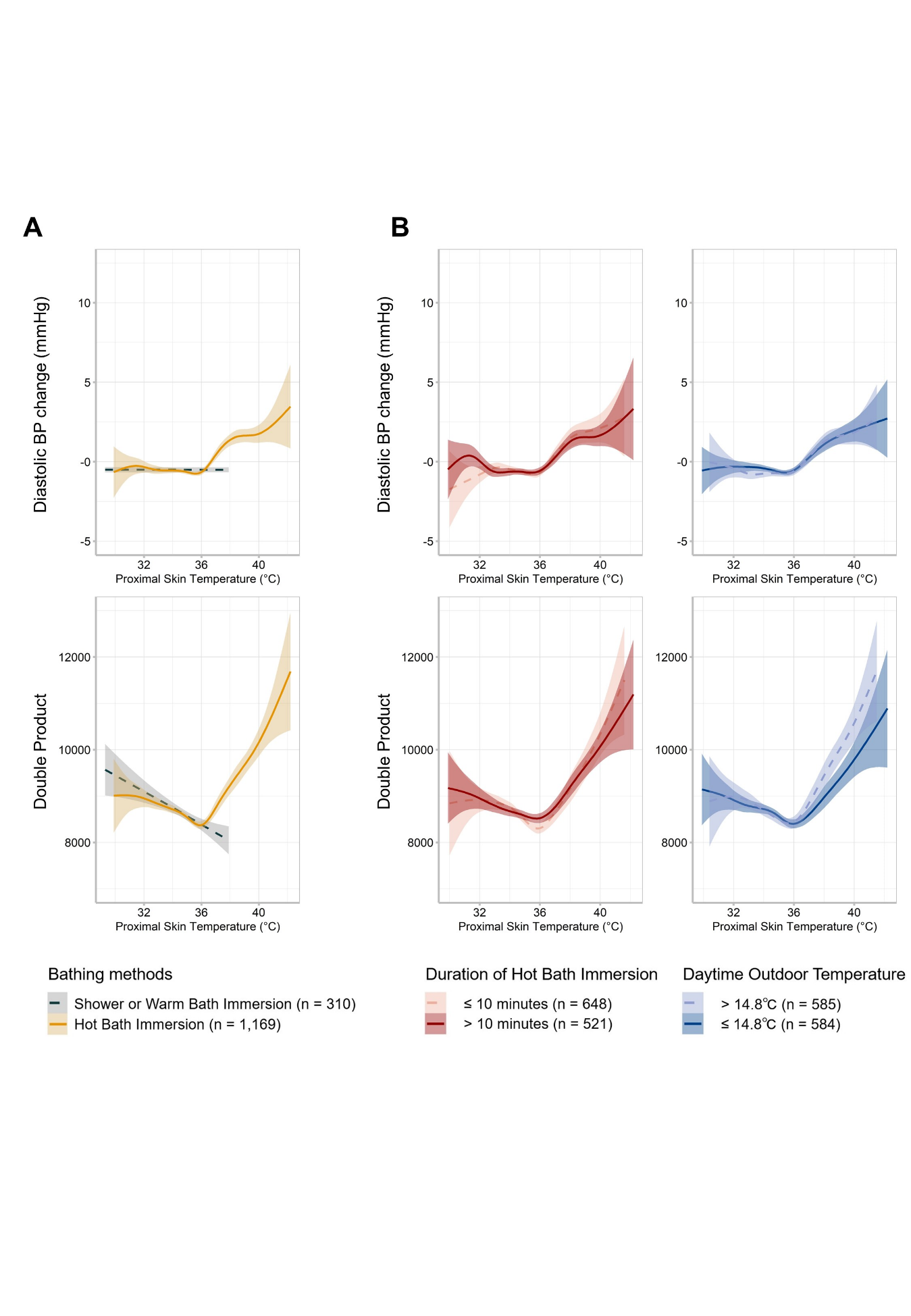
 **Additional file 2**. Association of proximal skin temperature with diastolic blood pressure and double product during bathing and the adjacent period

Participants were divided into two groups based on bathing method (A), duration of hot bath immersion (red line in B), and median daytime outdoor temperature (blue line in B). A generalized additive model was used to determine the association between proximal skin temperature and diastolic BP/double product. The shaded areas indicate the upper and lower 95% confidence intervals.

Diastolic BP, pulse rate, and proximal skin temperature were measured during bathing and 1–60 mins before and after bathing. Blood pressure was centered within the individual daytime mean.

BP, blood pressure.

**Additional file 3.** Association between proximal skin temperature and hemodynamic parameters during bathing and the adjacent period divided by four seasons, sex, and age groups


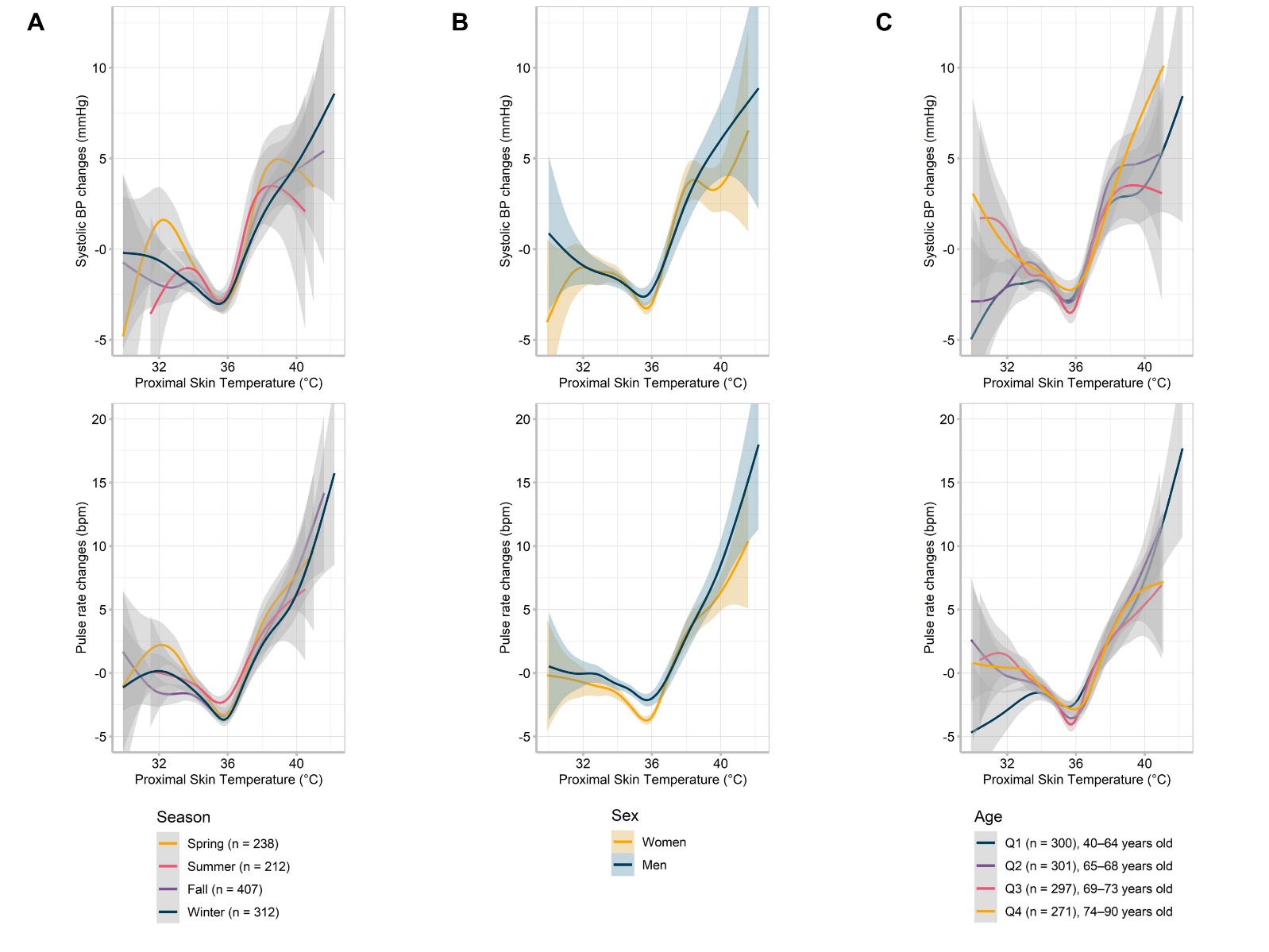


Participants were divided into four seasons (A), men and women (B), and age-quartile groups (C). A generalized additive model was used to determine the association between proximal skin temperature and systolic BP/pulse rate. The shaded areas indicate the upper and lower 95% confidence intervals.

Systolic BP, pulse rate, and proximal skin temperature were measured during bathing and 1–60 mins before and after bathing. Blood pressure was centered within the individual daytime mean.

BP, blood pressure.
